# Development and validation of a nomogram to predict the risk of death within 1 year in patients with non-ischemic dilated cardiomyopathy: a retrospective cohort study

**DOI:** 10.1038/s41598-022-12249-7

**Published:** 2022-05-20

**Authors:** Yuan Huang, Hai-Yan Wang, Wen Jian, Zhi-Jie Yang, Chun Gui

**Affiliations:** 1 Department of Cardiology, The First Affiliated Hospital of Guangxi Medical University, No 6 Shuangyong Road, Nanning, Guangxi 530021 People’s Republic of China; 2 Guangxi Key Laboratory Base of Precision Medicine in Cardio-Cerebrovascular Diseases Control and Prevention, No 6 Shuangyong Road, Nanning, Guangxi 530021 People’s Republic of China; 3 Guangxi Clinical Research Center for Cardio-Cerebrovascular Diseases, No 6 Shuangyong Road, Nanning, Guangxi 530021 People’s Republic of China

**Keywords:** Biomarkers, Diseases, Medical research, Cardiology, Cardiovascular biology

## Abstract

Predicting the chances mortality within 1 year in non-ischemic dilated cardiomyopathy patients can be very useful in clinical decision-making. This study has developed and validated a risk-prediction model for identifying factors contributing to mortality within 1 year in such patients. The predictive nomogram was constructed using a retrospective cohort study, with 615 of patients hospitalized in the First Affiliated Hospital of Guangxi Medical University between October 2012 and May 2020. A variety of factors, including presence of comorbidities, demographics, results of laboratory tests, echocardiography data, medication strategies, and instances of heart transplant or death were collected from electronic medical records and follow-up telephonic consultations. The least absolute shrinkage and selection operator and logistic regression analyses were used to identify the critical clinical factors for constructing the nomogram. Calibration, discrimination, and clinical usefulness of the predictive model were assessed using the calibration plot, C-index and decision curve analysis. Internal validation was assessed with bootstrapping validation. Among the patients from whom follow-up data were obtained, the incidence of an end event (deaths or heart transplantation within 1 year) was 171 cases per 1000 person-years (105 out of 615). The main predictors included in the nomogram were pulse pressure, red blood cell count, left ventricular end-diastolic dimension, levels of N-terminal pro b-type natriuretic peptide, medical history, in-hospital worsening heart failure, and use of angiotensin-converting enzyme inhibitors or angiotensin II receptor blockers. The model showed excellent discrimination with a C-index of 0.839 (95% CI 0.799–0.879), and the calibration curve demonstrated good agreement. The C-index of internal validation was 0.826, which demonstrated that the model was quite efficacious. A decision curve analysis confirmed that our nomogram was clinically useful. In this study, we have developed a nomogram that can predict the risk of death within 1 year in patients with non-ischemic dilated cardiomyopathy. This will be useful in the early identification of patients in the terminal stages for better individualized clinical decisions.

## Introduction

Non-ischemic dilated cardiomyopathy (NIDCM) is one of the most frequently occurring types of non-ischemic cardiomyopathy defined by the presence of contractile dysfunction and left ventricular dilatation. It is the main cause of systolic heart failure and also the most common cause of heart transplantations worldwide^1^. NIDCM is reported to occur in 5–7 cases per 100,000 person-years^2^ with an extremely high fatality rate^3^ and poor prognosis. And studies report a female to male ratio between 1:1.5 and 1:3^3,4^. The poor prognosis for most NIDCM patients puts a huge financial burden on individuals, families, and society.

Many factors affect the prognosis of NIDCM; these include genetics and family history, aetiology, gender, age, and presence of comorbidities such as renal failure, chronic obstructive pulmonary disease, etc.^5–9^. Several of these factors are of high predictive value for the prognosis of NIDCM^10^. In addition, other prognostic indicators related to heart failure (such as left ventricular size and ejection fraction^11^, and some new predictive indicators reflecting myocardial fibrosis may also be useful^12^. With improving healthcare facilities, better and more standardized drug treatments, and the development of new surgical treatment techniques, the prognosis of NIDCM patients has improved significantly in the past few decades^13,14^. For example, procedures like valve repair or valve replacement surgery for patients with valve insufficiency, catheter radiofrequency ablation and percutaneous left atrial appendage closure (LAAC) for patients with atrial fibrillation, cardiac resynchronization therapy (CRT) for patients with heart failure (whose left and right ventricular contractions are significantly out of sync), and implanting cardioverter defibrillators (ICD) for the prevention of sudden death have contributed to the improved prognosis observed in NIDCM patients^14^. However, these measures are not only expensive, but they are of little benefit to patients expected to have short survival times. The 2017 AHA (American Heart Association)/ACC (American College of Cardiology)/HRS (Heart Rhythm Society) Guidelines clearly state that ICD implantation is applicable for patients with expected survival times of > 1 year^15^. Patients with an expected survival period of < 1 year are considered to be in the terminal stages of the disease and are considered to be more suitable subjects for left ventricular assist device implantation or heart transplantation. Therefore, the assessment of the risk of death within 1 year for NIDCM patients is particularly important for weighing the benefits and risks of different life-saving procedures. Since heart failure is the most obvious feature of NIDCM, heart failure models are often used as alternative predictive tools^16,17^ to those predicting risk of death within 1 year. However, the common problems of current prediction models are that they are not accurate enough^8^, contain too many variables, and lack the key predictors of dilated cardiomyopathy, which is complicated to use and often requires formulas or software to operate^16,17^, the clinical application scenarios are limited. Most importantly, these models lack data on the Chinese population in basic population, and differences in demographic characteristics and disease spectrum are likely to limit the application of these models in the Chinese population. In addition, the heart failure scoring system commonly used in clinical practice has not been validated in the Chinese NIDCM population. To help with clinical decision-making in NIDCM patients, a specific 1-year mortality risk prediction model became an urgent need.

Nomograms are tools that have been useful in accurately predicting disease or complication risks^18,19^ in many situations. They are also a concise and intuitive way of presenting models. Based on data from the region's largest medical center, this study takes a more comprehensive consideration of the impact of various factors on prognosis and aimed to develop and verify a simple, practical, and accurate risk-prediction tool for patients with NIDCM. The nomogram we have developed can be used to assess the 1-year mortality risk in NIDCM patients to allow for better individualized clinical decisions.

## Results

### Participants

A total of 615 NIDCM patients of mean age 55 (46, 64) years were continuously enrolled in this study; of these, 468 were men and 147 were women. The information on patient status was gathered after 33.7 ± 24.2 months. According to the information collected from telephonic follow-up sessions and electronic medical records, all patients were divided into two groups: (1) the non-event group (510 cases), and (2) event group (all-cause death or heart transplantation occurring within 1 year, 105 cases). All baseline characteristics, including general information and results of physical examinations, blood biochemistry assays, treatment and drug regimens given, echocardiography, etc. are listed in Supplementary Table 1. There were no statistically significant differences in gender and age distributions between the event and the non-event groups. Compared with the non-event group, patients in the event group had longer medical histories, more complicated respiratory inflammation issues, worse heart failure grades, lower systolic blood and pulse pressures, lower red blood cell counts, higher levels of NT-proBNP and inflammation indicators, higher levels of myocardial enzymes, worse liver and kidney functions, larger left ventricles, and more emergency hospital incidents. Furthermore, more patients in the event group unconditionally used ACEIs or ARBs than those in the non-event group (all P < 0.01).

### Performance of the MAGGIC in the NIDCM cohort

We validated the MAGGIC score scale using data from the NIDCM cohort. Indeed, surviving and non‐surviving patients were differentiated by mortality risk calculated by the analysed prognostic score (Table 1). However, the scale substantially underestimated mortality risk for surviving patients. The diagnostic ability of the MAGGIC scale was general, and the area under the curve of 1, 2 and 3 year mortality was respectively 0.684, 0.709, and 0.691.

**Table 1:** Comparison of calculated mortality risk for non‐survivors and survivors of non-ischemic dilated cardiomyopathy patients.

	Non‐survivors (n = 221)	Survivors (n = 394)	*P* value
Follow‐up time (month)	18.7 ± 19.7	42.1 ± 22.5	< 0.001
**1‐year mortality**			
Outcome of study population, n (%)	105(17.1%)	510(82.9%)	
Mortality risk by MAGGIC (%)	16.7 ± 9.5	11.5 ± 6.5	< 0.001
**3‐year mortality**			
Outcome of study population, n (%)	183(44.7%)	226(55.3%)	
Mortality risk by MAGGIC (%)	35.1 ± 15.5	25.1 ± 11.5	< 0.001

*MAGGIC* meta‐analysis global group in chronic heart failure, *MUSIC* MUerte Subita en Insuficiencia Cardiaca. Follow‐up time was presented as mean ± SD. The mortality in study population was presented as n (%). Predicted mortality was calculated based on the website (www.heartfailurerisk.org).

### Nomogram development

**Figure 1:**
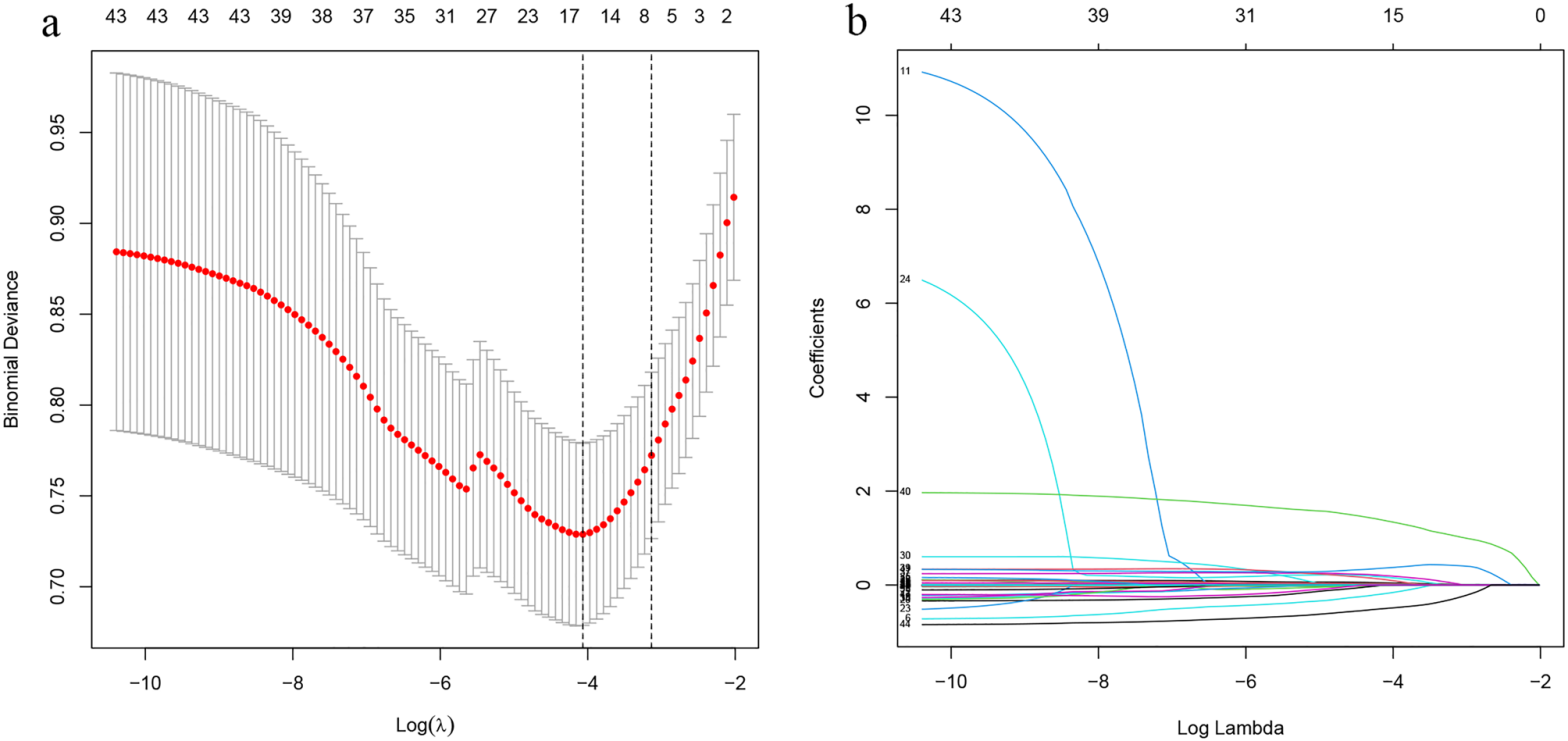
Selection of risk factors contributing to mortality within 1 year in NIDCM patients using the LASSO regression model. (**a**) Optimal parameter (lambda) selection in the LASSO model used five-fold cross-validation via minimum criteria. The partial likelihood deviance (binomial deviance) curve was plotted against log(lambda). Dotted vertical lines were drawn at the optimal values by using the minimum criteria and a SE of 1 for the minimum criteria (the 1-SE criteria). (**b**) LASSO coefficient profiles of the 42 features. A coefficient profile plot was generated against the log(lambda) sequence.

We plugged the variables which differed significantly (P < 0.05) between the event and non-event groups into the LASSO regression to further screen for appropriate risk prediction indicators. Then the variables were reduced to 16 potential predictors (Fig. 1). These predictors included body mass index, systolic pressure, pulse pressure, red blood cell count, neutrophil to lymphocyte ratio, total serum cholesterol levels, serum chlorine levels, international normalized ratio, aspartate aminotransferase levels, NT-proBNP levels, left ventricular end-diastolic dimension (LVDd), medical history, presence of respiratory inflammation, in-hospital worsening heart failure, Dopamine Injection, and use of ACEIs or ARBs.

**Figure 2:**
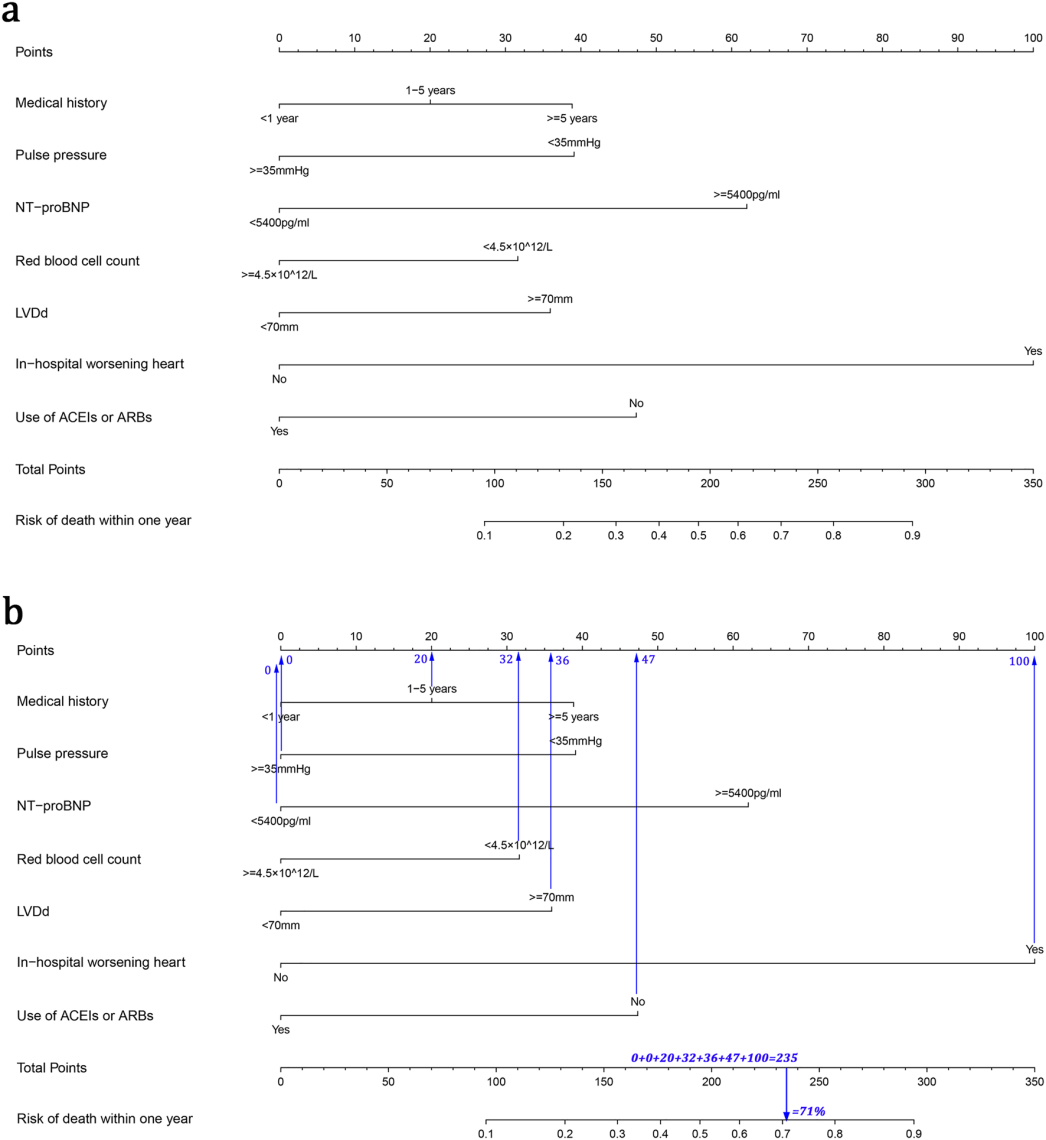
Nomogram for assessing the risk of death within 1 year in NIDCM patients. (**a**) Complete nomogram. (**b**) How to use the nomogram.

To make the model simpler and more practical, we transformed the continuous data in the 16 potential predictors above into count data based on optimal cut-off points and clinical significance (The detail was shown in Supplementary Table 2). Following this, the 16 variables selected through the LASSO regression were included in a non-conditional binary logistic regression. Multivariate logistic regression analysis revealed that pulse pressure (odds ratio (OR) 0.533, 95% confidence interval (CI) 0.289–0.982, P = 0.044), red blood cell count (OR 0.499, 95% CI 0.296–0.841, P = 0.009), NT-proBNP (OR 2.904, 95% CI 1.633–5.166, P < 0.001), LVDd (OR 2.337, 95% CI 1.349–4.049, P = 0.002), length of medical history (≥ 5 years) (OR 2.127, 95% CI 1.082–4.183, P = 0.029), in-hospital worsening heart failure (OR 6.031, 95% CI 2.284–15.926, P < 0.001), and use of ACEIs or ARBs (OR 0.408, 95% CI 0.228–0.731, P = 0.003) were independent risk factors for predicting the risk of mortality within 1 year in NIDCM patients (Table 2). The nomogram was drew based on the above 7 factors (Fig. 2a).

**Table 2:** Results of Logistic regression.

Variables	Univariate analysis			Multivariate analysis		
	*®*	Odds ratio (95% CI)	*P* value	*®*	Odds ratio (95% CI)	*P* value
Body mass index	− 0.733	0.480 (0.296–0.779)	0.003	− 0.532	0.588 (0.331–1.043)	0.069
Systolic pressure	− 0.745	0.475 (0.310–0.726)	0.001	− 0.143	0.867 (0.472–1.593)	0.646
Pulse pressure	− 0.716	0.489 (0.319–0.748)	0.001	− 0.629	0.533 (0.289–0.982)	0.044
Red blood cell count	− 0.811	0.444 (0.290–0.680)	< 0.001	− 0.696	0.499 (0.296–0.841)	0.009
NLR	1.196	3.306 (2.135–5.119)	< 0.001	0.117	1.124 (0.626–2.019)	0.695
Total cholesterol	− 0.706	0.494 (0.321–0.759)	0.001	− 0.218	0.804 (0.470–1.375)	0.426
Serum chlorine	− 1.612	0.199 (0.118–0.337)	< 0.001	− 0.547	0.579 (0.280–1.197)	0.140
International normalized ratio	0.942	2.566 (1.527–4.312)	< 0.001	0.122	1.130 (0.572–2.231)	0.725
Aspartate aminotransferase	0.933	2.541 (1.656–3.899)	< 0.001	0.48	1.616 (0.944–2.767)	0.080
NT-proBNP	1.761	5.816 (3.601–9.394)	< 0.001	1.066	2.904 (1.633–5.166)	< 0.001
LVDd	0.893	2.442 (1.577–3.781)	< 0.001	0.849	2.337 (1.349–4.049)	0.002
Medical history(< 1 year)			< 0.001			0.090
Medical history(1–5 years)	0.768	2.154 (1.303–3.562)	0.003	0.411	1.508 (0.802–2.836)	0.202
Medical history(≥ 5 years)	1.087	2.966 (1.719–5.119)	< 0.001	0.755	2.127 (1.082–4.183)	0.029
Respiratory inflammation	0.928	2.530 (1.650–3.880)	< 0.001	0.394	1.483 (0.860–2.557)	0.157
In-hospital worsening heart failure	2.696	14.815 (6.831–32.13)	< 0.001	1.797	6.031 (2.284–15.93)	< 0.001
Dopamine Injection	1.493	4.452 (2.860–6.933)	< 0.001	0.169	1.185 (0.649–2.161)	0.581
ACEIs or ARBs	− 1.357	0.258 (0.160–0.414)	< 0.001	− 0.897	0.408 (0.228–0.731)	0.003

. *NLR* neutrophil to lymphocyte ratio, *NT-proBNP* N terminal pro B type natriuretic peptide, *LVDd* left ventricular end diastolic dimension, *ACEIs* angiotension converting enzyme inhibitors, *ARBs* angiotensin receptor blockers, *CI* confidence interval. *®* is the regression coefficient.

The nomogram that we have developed can be used as demonstrated below:

Consider an NIDCM patient who was admitted to the hospital with acute heart failure; after receiving occurring of in-hospital worsening heart failure (100 points), the patient’s condition stabilised. The length of the patient’s medical history was 3 years (20 points), blood pressure was 93/55 mmHg, pulse pressure was 38 mmHg (0 points), NT-proBNP level was 3600 pg/ml (0 points), red blood cell count was 4.2 × 10^12^/l (32 points), LVDd was 75 mm (36 points), and the patient was not on ACEIs or ARBs due to low blood pressure (47 points). In summary, the patient had a total score of 235 points, and the corresponding predicted risk of mortality within 1 year was 0.71 (71%) (Fig. 2b). In terms of overall morbidity, the patient had a high risk of dying within 1 year.

### Nomogram validation

**Figure 3:**
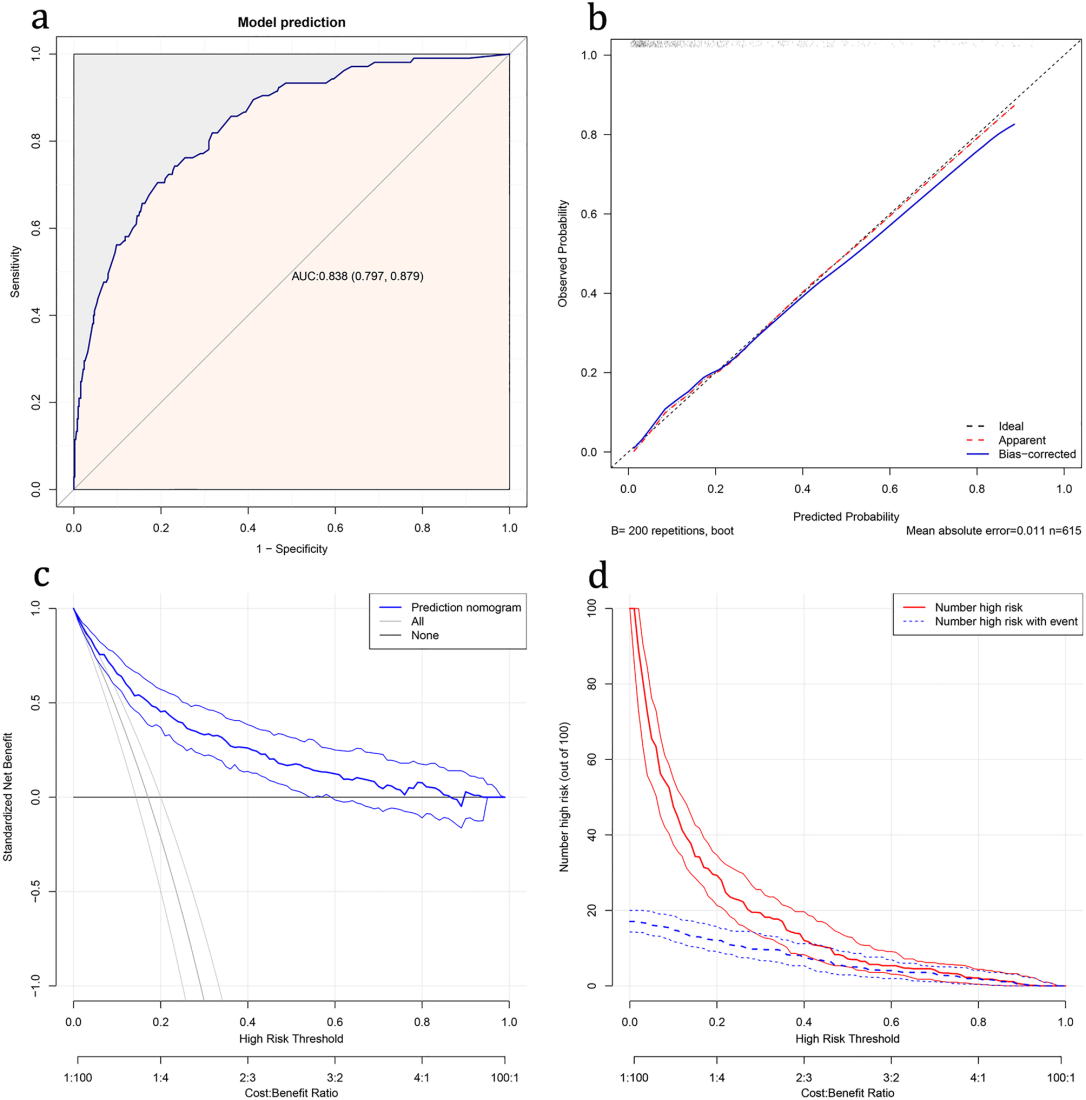
Nomogram validation. (**a**) ROC curve for the nomogram. (**b**) Calibration curve for the nomogram. The x-axis represents the predicted 1-year mortality risk. The y-axis represents the actual confirmed 1-year mortality. The diagonal dotted line represents a perfect prediction by an ideal model. The solid line represents the performance of the nomogram, of which a closer fit to the diagonal dotted line represents a better prediction. (**c**) Decision curve analysis for the nomogram. The y-axis measures the standardized net benefit. The blue line represents the nomogram and its 95%CI. The thin solid line represents the assumption that all patients die within 1 year. The thick solid line represents the assumption that no patients die within 1 year. (**d**) Clinical impact curve for the nomogram. The solid red line represents the predicted number of people and 95% CI judged as high risk by the model at different risk thresholds. The dotted blue line represents the actual number of high-risk people and 95% CI at different risk thresholds.

The validation of the model was based on discrimination and calibration. In Fig. 3a, we generated the receiver operating characteristic (ROC) curve of predicted probability and calculated the AUC was 0.838. We also calculated the C-index to evaluate the model’s discrimination performance. The C-index was 0.839 (95% CI 0.799–0.879), and the C-index of internal validation was 0.826, which further demonstrated that the model was efficacious. For verification of calibration, we conducted the Hosmer–Lemeshow test, for which the model exhibited a P value of 0.901 (P > 0.05); we also generated a calibration curve to further illustrate the agreement between predicted mortality and actual mortality (Fig. 3b). The decision curve to guide clinical applications of the nomogram is presented in Fig. 3c. The decision curve shows that at threshold probabilities of > 5% and < 80%, using the nomogram to predict 1-year mortality risks will reap the net clinical benefit. All the results explained above have verified the high predictive ability of our nomogram. The clinical impact curve came from the clinical decision curve, which showed the estimated number of people at each risk threshold who would be declared high risk and visually showed the proportion of cases (true positive) (Fig. 3d).

### Sensitivity analysis

**Figure 4:**
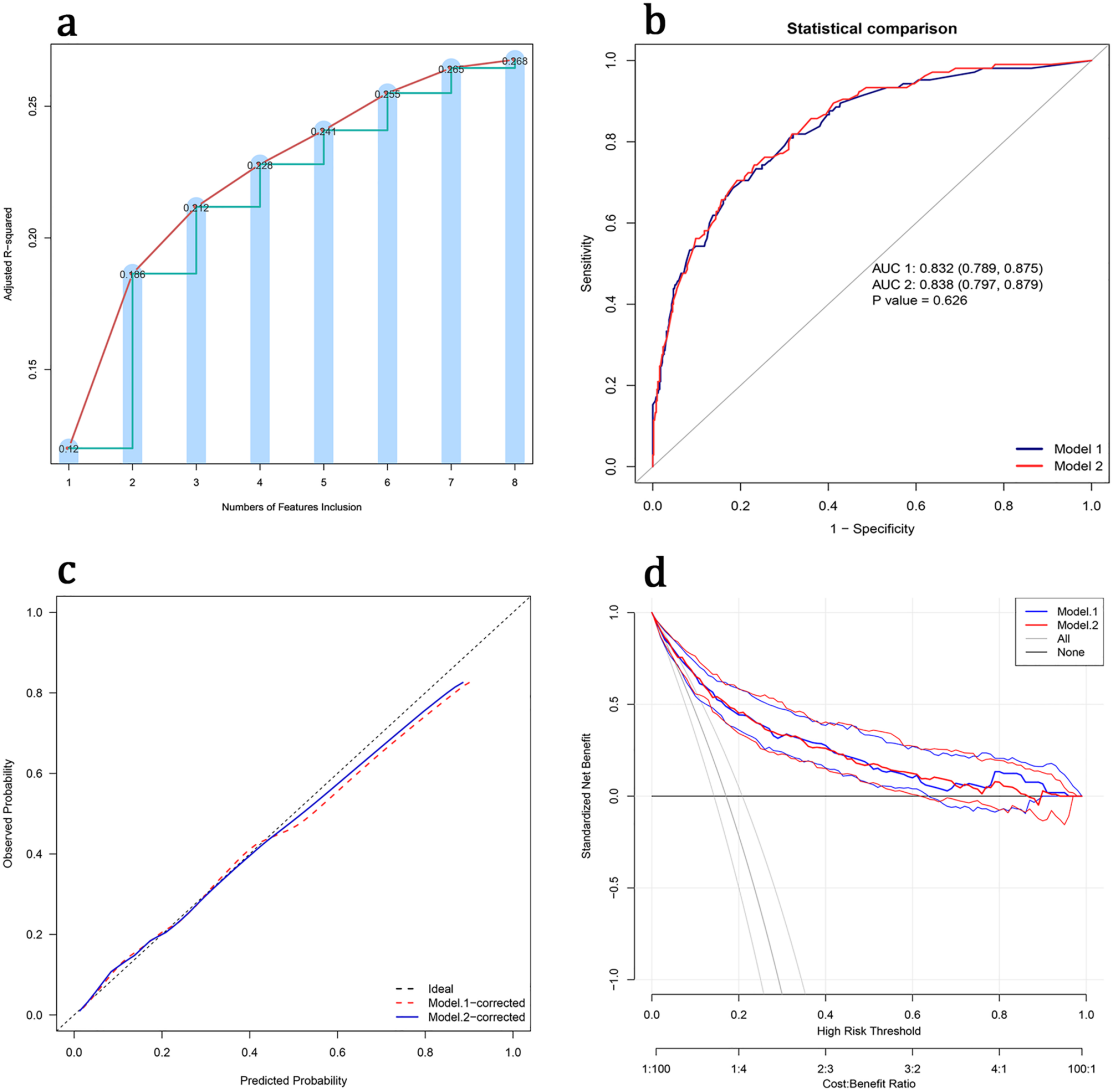
Comparison of different variable screening methods. (**a**) The abscissa represents the number of variables included, and the ordinate represents the value of adjusted R-square; when the number of variables is 8, the maximum adjusted R-square is 0.268. (**b**) Comparison of ROC curves between model 1 and model 2. (**c**) Comparison of calibration curves between model 1 and model 2. (**d**) Comparison of decision curves between model 1 and model 2.

Firstly, we changed variable screening methods to compare whether different methods can screen out a better combination of variables. We adopted the Best Subset Selection^20^, selected the variable combination of maximum adjusted R squared (Fig. 4a): systolic pressure, NT-proBNP, neutrophil to lymphocyte ratio, aspartate aminotransferase, LVDd, Dopamine Injection, use of ACEIs or ARBs, in-hospital worsening heart failure. The continuous variables were classified according to the optimal truncation value (Supplementary Table 2), and then the logistic regression was used to construct the model (Model 1). The ROC curve, calibration curve, and clinical decision curve were used to compare model 1 with the original model (Model 2) (Fig. 4b–d). The results show that there is no significant difference between the two models, but the original model contains fewer variables and is more practical.

**Figure 5:**
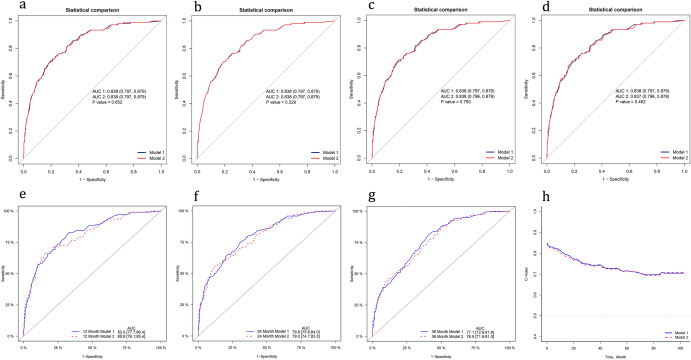
Comparison with the original model(model 1) after adding variables(model 2). (**a**) Added "MRA" to the model. (**b**) Added "Implantable cardiac devices" to the model. (**c**) Added "Ventricular tachycardia/fibrillation" to the model. (**d–h**) Added "MRA + Implantable cardiac devices + Ventricular tachycardia/fibrillation" to the model.

Secondly, considering that mineral corticoid receptor antagonist (MRA), history of implantable cardiac devices and ventricular tachycardia/fibrillation may be closely related to the mortality of NIDCM patients, we added these variables into the model and compared them through ROC curves and C-index. We found that the area under the ROC curves showed no significant difference regardless of whether these three variables were added into the model one by one (Fig. 5a–c) or at the same time (Fig. 5d). We also compared the ROC curves for 1-year, 2-year, and 3-year mortality with the simultaneous inclusion of these three variables in the model, however, the model performance did not improve (Fig. 5e–g). In addition, with the extension of follow-up time, the change in the C-index of the two models showed a synchronous decline trend, and there was no difference between them (Fig. 5h).

**Figure 6:**
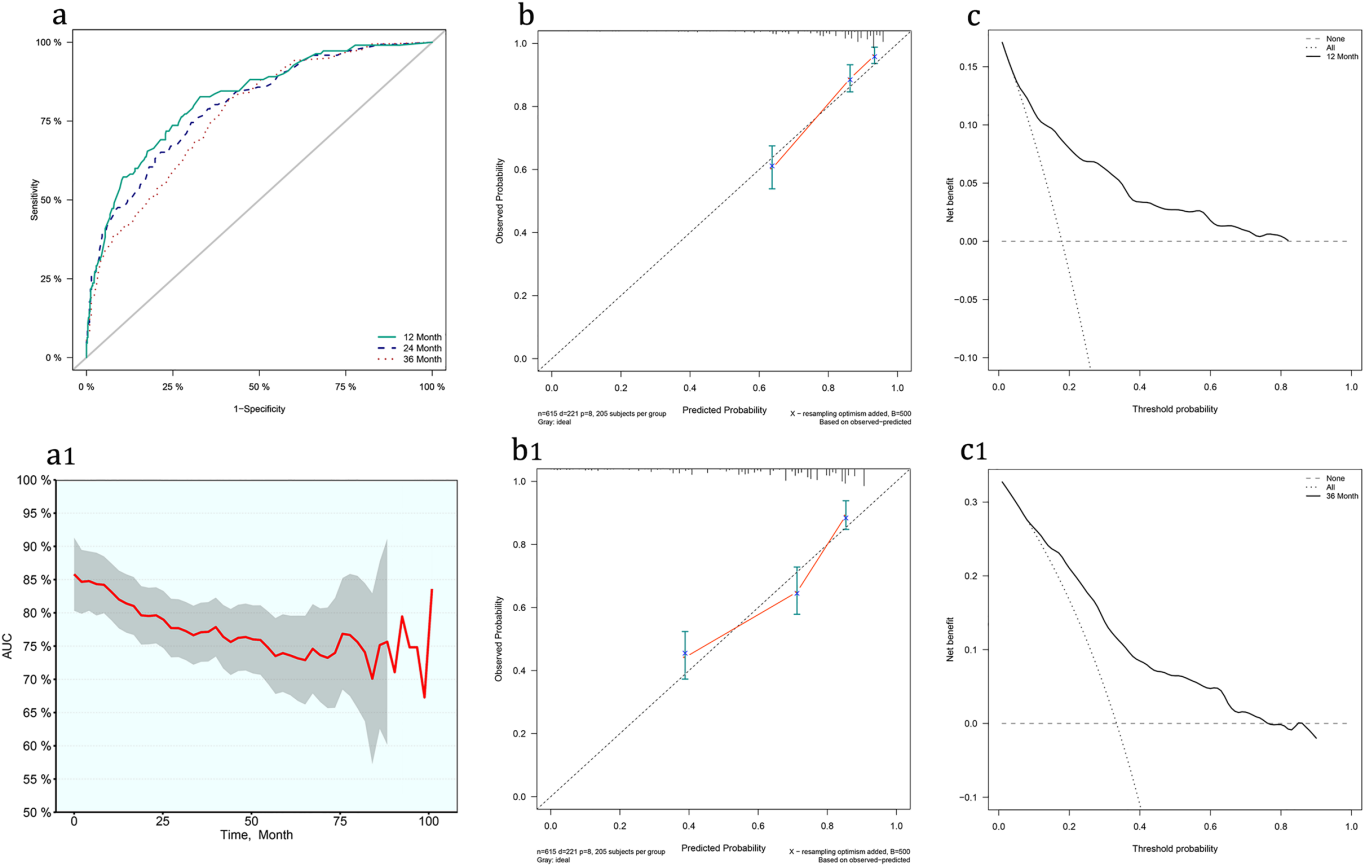
Comparison of models with different regression methods. (**a**,**a1**) ROC curve and AUC at different times. (**b**,**b1**) Calibration curves for 1- and 3-year mortality. (**c**,**c1**) Decision curves for 1- and 3-year mortality.

We included the follow-up time into the model and analyzed the data again by the COX regression. The AUC of 1-year, 2-year, and 3-year mortality were 0.82, 0.80, and 0.77, respectively (Fig. 6a). The calibration curve and decision curve performed well (Fig. 6b,b1,c,c1). It shows that the combination of variables selected is excellent and reliable. However, with the extension of follow-up time, not only did the AUC gradually decrease but also the confidence interval significantly expanded (Fig. 6a1). It suggests that the subsequent results are not stable, which may be related to the increase of truncated data. Therefore, based on the current data, it is necessary to be cautious to use the COX regression model to predict medium and long-term prognosis.

**Figure 7:**
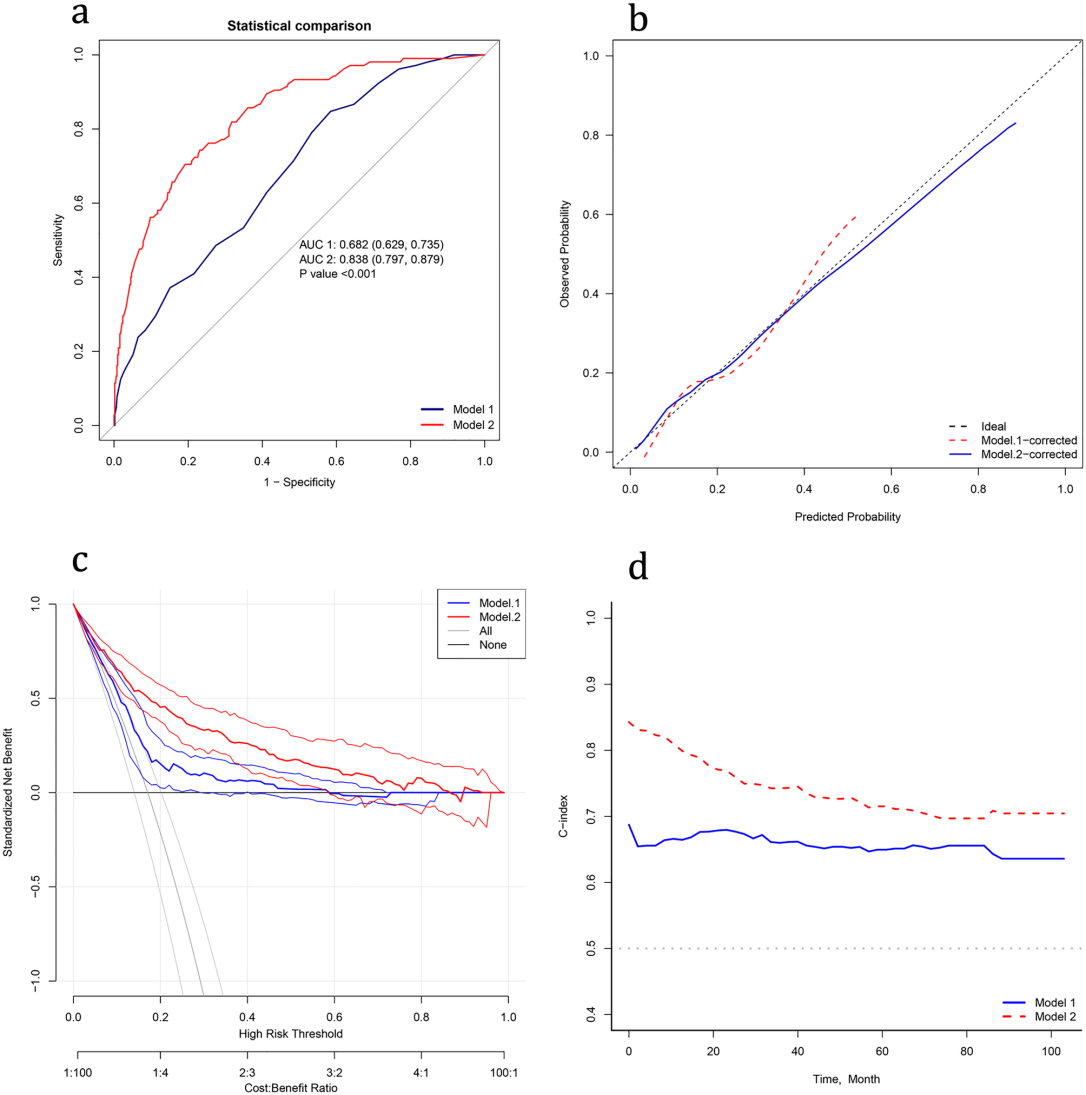
Comparison between our nomogram(model 2) and the MAGGIC score scale(model 1). (**a**) Comparison of ROC curves between model 1 and model 2. (**b**) Comparison of calibration curves between model 1 and model 2. (**c**) Comparison of decision curves between model 1 and model 2. (**d**) Comparison of C-index between model 1 and model 2.

Finally, we compared the MAGGIC score scale with the nomogram we constructed. The results show that our nomogram is significantly superior to the MAGGIC score scale in predicting 1-year mortality in the ROC curve, calibration curve, and clinical decision curve (Fig. 7a–c). In addition, the C-index of medium and long-term prognosis was significantly higher than the MAGGIC score scale (Fig. 7d), but this result still needs more data support.

## Discussion

The Alignment Diagram, also known as Nomogram Diagram, is based on multi-factor regression analysis, integrating multiple prediction indicators and drawing them in a certain proportion on the same plane with graduated line segments, to express the relationship between variables in the prediction model. Assign scores to the value level of each variable in the model, and then add the scores to get the total score. Finally, the predicted value of the individual outcome event is calculated through the function conversion relationship between the total score and the probability of the outcome event. Due to its user-friendly digital interface, high accuracy, and easily understood outputs, nomograms are widely used prognostic devices in medicine (especially in oncology) to aid clinical decision making^21,22^. We have, for the first time, developed a nomogram for NIDCM patients to predict the risk of mortality within 1 year. We developed and validated this prediction tool for determining the risk of mortality within 1 year for NIDCM patients based on 7 key predictors screened by LASSO and uni- and multivariate logistic regression analyses. Internal verification also demonstrated that our nomogram had good discrimination and calibration power.

Since knowing the prognosis is very important for making clinical decisions in NIDCM patients, our model can help doctors and caregivers to choose the best possible treatment options for patients. Because of a failure to detect and treat NIDCM early on, many patients have poor cardiac function. In clinical work, doctors are accustomed to evaluating the severity and prognosis of NIDCM by indicators of heart failure (such as the NYHA (New York heart association) functional classification and left ventricular ejection fraction (LVEF))^23,24^, which are at best, very crude and subjective estimations given the complexity of the disease; due to this, the accuracies of prognoses made for NIDCM patients is usually poor^25^. For example, for the patients with NIDCM combined with atrial fibrillation, the measurement of the EF value is not accurate, and any prognoses made based on this measurement will be inaccurate. Dziewiecka et al.^8^ found that in the NIDCM population, the prognostic accuracy of the most frequently applied heart failure prognostic scales were suboptimal, varying between 60 and 80%, which is consistent with our conclusion from the verification of the MAGGIC score scale. To effectively and accurately identify terminal NIDCM patients and formulate corresponding individualized treatment strategies based on clinical real-world data, we constructed this prognostic nomogram, which can easily quantify the risk of an NIDCM patient dying within 1 year.

Many factors affect the prognosis of patients with NIDCM. In our study, we have found that pulse pressure, red blood cell count, NT-proBNP levels, LVDd, length of medical history (≥ 5 years), in-hospital worsening heart failure, and use of ACEIs or ARBs were independently associated with the risk of mortality within 1 year for NIDCM patients. Levels of NT-proBNP have been widely used in clinical practice as markers of heart failure as the levels of this protein have a linear relationship with the degree of heart failure. Many studies have shown that levels of NT-proBNP are significantly related to the LVEF^24^ and NYHA functional classification. Therefore, NT-proBNP levels are used in many heart failure models^23,26^ to predict the prognosis of patients with heart failure. In our model, the level of NT-proBNP in blood was an independent risk factor for the risk of mortality within 1 year. After adjusting for the influence of related factors, the risk of death within 1 year in patients with NT-proBNP ≥ 5400 pg/ml was about 2.9 times that of patients NT-proBNP levels are < 5400 pg/ml. The value of LVDd is also one of the key factors affecting the prognosis of NIDCM patients. Previous studies have shown that severe left ventricular dilatation additively increased the risk of sudden cardiac death (SCD). Left ventricular diameter may also contribute to risk stratification for SCD independent of the LVEF^27^. In addition, the size of the LVDd is often combined with LVEF to evaluate the recovery of cardiac function^28,29^. As heart function decreases, stroke volume reduces and systolic pressure also decreases in patients with NIDCM. At the same time, due to the compensation mechanisms of the heart, the heart rate increases, which also raises diastolic pressure; this causes the pulse pressure (which is the difference between systolic and diastolic pressures) to decrease. Therefore, the lower the pulse pressure, the worse the heart function. The haemoglobin in red blood cells is crucial for oxygen transport in the blood. Reductions in the numbers of red blood cells can seriously affect the oxygen-carrying capacity of blood and NIDCM patients with low red blood cell counts are at a higher risk of short-term mortality. The 2016 list of criteria formulated by the International Society for Heart and Lung Transplantation mentions that the peak oxygen consumption during cardiopulmonary exercise testing can be used as an effective measure for the requirement of a heart transplant (Class I recommendation, Level B evidence)^30^. This measure of the maximum oxygen consumption capacity of the human body during extreme exercise essentially reflects the heart reserve function. Therefore, the effect of red blood cell count on the short-term prognosis of dilated heart disease may be related to the heart–oxygen reserve mechanism^31,32^. Short-term mortality risk is also known to be directly proportional to the length of medical history. The risk of death within 1 year for a patient with a medical history of < 1 year as compared to that of a patient with a 1–5 year-long medical history or one with a medical history of ≥ 5 years is 1.5 and 2.1 times lower, respectively. We all know that without effective intervention, dilated cardiomyopathy often shows progressive development. All the patients included in this study were hospitalized for the first time in our hospital, and most patients had failed to conduct standardized and continuous treatment before. Therefore, the longer the medical history, the more serious the disease. When heart function deteriorates rapidly, the body is in an acute ischemic and hypoxia state, which quickly increases the burden on the heart. Moreover, the damage to the heart is often serious and irreversible as cardiomyocytes are non-renewable cells. Therefore, the incidence of in-hospital worsening heart failure can have an important predictive value for the short-term prognosis of NIDCM patients. Finally, the use of ACEIs or ARBs are also key factors affecting the prognosis of NIDCM patients; this has been confirmed by a large number of previous studies^29,33^.

Overall, we have created a nomogram for assessing the risk of mortality within 1 year for NIDCM patients by using several common predictors in clinical practice; this model has been verified as having a good predictive value. Sensitivity analysis of our original model was performed by using different variable screening methods, adding clinically key variables, different model-building methods, and comparing with an external heart failure score scale. The results showed that the model was stable and reliable. The nomogram chart is a useful supplementary tool for clinical work and has been shown to affect clinical decision-making positively. For patients with high predicted 1-year mortality, physicians will not be inclined to implant ICD, treat atrial fibrillation with radiofrequency ablation, or perform LAAC. Instead, more effective recommendations, such as heart transplantation or left ventricular assist devices implantation, will be given to help patients increase their chances of survival. Therefore, it has an important guiding significance in improving prognosis and reducing unnecessary medical expenditure.

Although we have comprehensively considered various factors to construct a simple short-term mortality risk model with good reliability, our study suffers from several limitations. Firstly, the population in our study was not on behalf of all Chinese patients with NIDCM, and patients without access to treatment were not incorporated into study. In addition, this was a retrospective study, due to which, selection bias could not be avoided. To reduce the effect of this bias, we set very strict inclusion and exclusion criteria and collected adequate numbers of clinical samples to accurately reflect the actual conditions of event occurrence. However, prospective studies to provide more evidence of the usefulness of our model are still required. Secondly, our risk factor analysis did not cover all potential factors that affected the short-term prognosis of NIDCM patients. Some possible factors such as the degree of myocardial fibrosis^12,34^ were not thoroughly investigated, and our model also does not account for the effects of other causes of NIDCM and some key genes known to cause/affect NIDCM patients. Thirdly, all the data for our prediction model were obtained from a single hospital. Although the robustness of our nomogram was examined sufficiently with internal validation, we will still need to test the nomogram with data from other hospitals for external validation. Fourthly, we need more data and a longer follow-up time for the medium and long-term prognosis of NIDCM, and we will continue to advance this research in the future.

## Methods

### Patients

**Figure 8:**
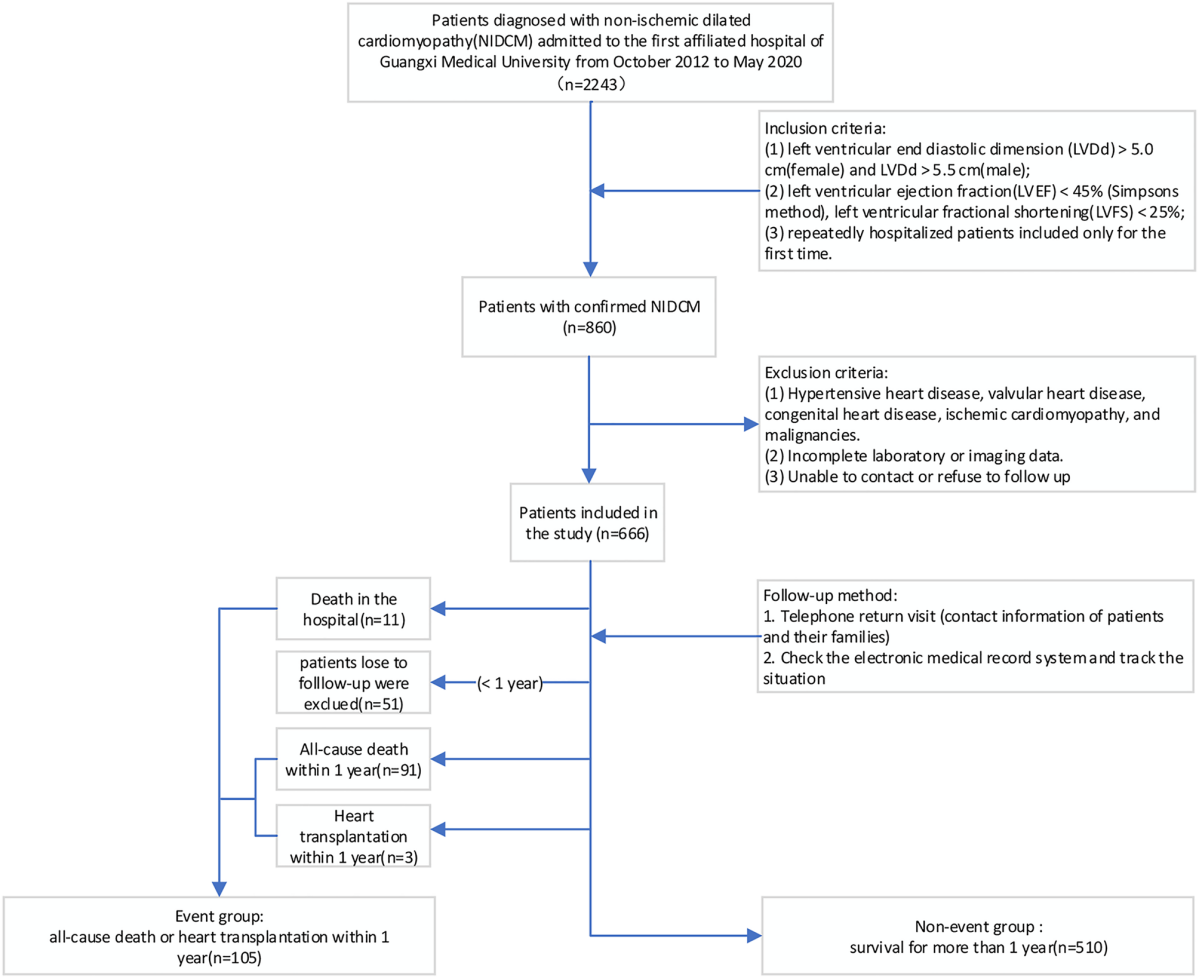
Flow chart showing the process of patient recruitment for the study population.

The cohort of our study was identified following an evaluation of the medical records system, from October 2012 to May 2020. We diagnosed patients with NIDCM based on echocardiography, imaging, and clinical symptoms. The inclusion and exclusion criteria are in line with the Guidelines for the Diagnosis and Treatment of Dilated Cardiomyopathy in China^35^, with objective evidence of ventricular enlargement and reduced myocardial contractility. The process of patient recruitment for the study population is shown in Fig. 8. Except for patients who had an end-point event (death or heart transplant within 1 year) or were lost to follow-up, data for more than 1 year were collected on all selected patients through follow-up phone calls and by accessing their electronic medical records.

We estimated the lower sample size based on a binary outcome event; this was obtained as a value 5–10 times that of the variables included in the model; we further estimated the total sample size based on the incidence rate of end-point event to match the scale of the study. To ensure the reliability of the data, we excluded patients who had incomplete laboratory or imaging data.

### Data collection

The demographics and clinical characteristics of each patient, including general information on physical examination, blood biochemistry, echocardiography, and drug treatment regimens were obtained from electronic medical records. A total of 73 variables were included in this study. General information and physical examination data were collected within the first 8 h of hospitalization. Blood samples were collected within the first 24 h of hospitalization; all blood samples were sent to the inspection centre of the First Affiliated Hospital of Guangxi Medical University for biochemical assays. Through the electronic medical record system, we reviewed the time of biochemical blood sample collection and reporting, collected the first data within 24 h of admission, and we excluded repeated measures after the intervention. In addition, medical records with missing key laboratory indicators were excluded. Echocardiographic data were collected within the first 48 h of hospitalization. For patients on whom tests were repeated, only the first results at the time of hospitalization were utilised. Atrial fibrillation was diagnosed by electrocardiography, and pulmonary hypertension was diagnosed via transthoracic echocardiography performed by an experienced sonographer. Pulmonary artery pressures were estimated based on the tricuspid regurgitation pressure difference. Respiratory inflammation was defined as bronchitis or pneumonia with objective evidence of inflammatory infection. History of implantable cardiac devices defined as implantable cardioverter–defibrillator or cardiac resynchronization therapy before or during hospitalization. Records of the diagnosis, treatment regimens during the hospitalization period, and the treatment process were collected and combined with similar data obtained during follow-up sessions to understand the patient’s medication regimens after discharge. Meta‐Analysis Global Group in Chronic Heart Failure (MAGGIC)^16^ is one of the most commonly used heart failure prognostic scales. It was developed based on data from 30 cohort studies and contained a total of 13 variables: Age, Gender, Diabetes, COPD, Heart failure diagnosed within the last 18 months, Current smoker, NYHA Class, Receives beta blockers, Receives ACEI/ARB, BMI, Systolic blood pressure, Creatinine, and Ejection fraction. We collected information on these parameters and calculated each patient's risk score and 1-year and 3-year risk of death through the website (www.heartfailurerisk.org). In-hospital worsening heart failure defined as worsening heart failure symptoms and signs requiring an intensification of therapy during hospitalization^36^. We defined the endpoint as all-cause death or heart transplantation occurring within 1 year from the first hospitalization.

### Statistical analysis

Statistical analysis was performed using the Statistical Package for the Social Sciences 20.0 (SPSS Inc., Armonk, NY, USA) and R software (Version 4.0.4; https://www.r-project.org). Normality test found that the data in the study were not normally distributed. Therefore, the continuous variables were expressed as medians (quartiles), and categorical variables were expressed as frequencies (percentages). All continuous variables were analysed using the Mann–Whitney U test, and all categorical variables were analysed using the chi-square test or Fisher’s exact test. All variables in the above tests that varied significantly (P value < 0.05) across the test groups were identified as potential risk factors and used for further analysis. These variables were further screened using the LASSO regression, which is used for the reduction in high-dimensional data^37^. Non-zero coefficient variables were chosen in the LASSO regression model. The results of these analyses were used to select optimal predictive features in the risk factors identified in patients with NIDCM^38^. Uni- and multivariate logistic regression analyses were used to confirm independent risk factors that could predict the risk of death within 1 year in patients with NIDCM. Finally, a prediction model was established using these independent risk factors, which was evaluated for discrimination and calibration performance. The discrimination performance of a predictive model refers to its ability to distinguish between patients who have undergone events from those who have not. Generally, area under receiver operating characteristic curve (AUC) > 0.75 indicates that a model shows good discrimination performance^39^. To better quantify the discrimination performance of the predictive model, Harrell's C-index was also measured. The bootstrap method (1000 bootstrap resamples) was used for internal verification to avoid potential overfitting, following which, a corrected C-index was calculated^40^. Calibration curve was plotted to evaluate the calibration of the predictive model. Decision curve analysis was conducted to determine the clinical usefulness of the model by quantifying the net benefits at different threshold probabilities in NIDCM patients^41^. The net benefit was calculated by subtracting the proportion of all false positives from the proportion of true positives and by weighing the relative harm of forgoing interventions compared with the negative consequences of an unnecessary intervention^42^.

### Ethics approval and consent to participate

The authors are accountable for all aspects of the work in ensuring that questions related to the accuracy or integrity of any part of the work are appropriately investigated and resolved. The study was conducted in accordance with the Declaration of Helsinki (as revised in 2013). This study was approved by the Ethics Committee of the first affiliated Hospital of Guangxi Medical University; written informed consent was obtained from the patient himself or his close relatives.

## Supplementary Information

Supplementary Information.

## Data Availability

The datasets generated during and/or analysed during the current study are not publicly available due to the data belong to the hospital database but are available from the corresponding author on reasonable request.

## Abbreviations

ACEIs: Angiotensin-converting enzyme inhibitors
APTT: Activated partial thromboplastin time
ARBs: Angiotensin II receptor blockers
AUC: Area under curve
α-HBD: Alpha-hydroxybutyrate dehydrogenase
CI: Confidence interval
CRT: Cardiac resynchronization therapy
ICD: Implanting cardioverter defibrillators
LAAC: Left atrial appendage closure
LAD: Left atrium anteroposterior dimension
LASSO: Least absolute shrinkage and selection operator
LVDd: Left ventricular end diastolic dimension
LVEF: Left ventricular ejection fraction
LVFS: Left ventricular fractional shortening
NIDCM: Non-ischemic dilated cardiomyopathy
NT-proBNP: N terminal pro B type natriuretic peptide
NYHA: New York Heart Association
OR: Odds ratio
ROC: Receiver operating characteristic
SCD: Sudden cardiac death
SE: Standard error
