# Supplementary material for: Development and validation of a nomogram to predict the risk of death within 1 year in patients with non-ischemic dilated cardiomyopathy: a retrospective cohort study
Source: Sci Rep. 2022 May 20;12:8513. doi: 10.1038/s41598-022-12249-7 (PMC9123170; doi:10.1038/s41598-022-12249-7)
Supplement: Supplementary file 1 — Supplementary Information. [file 41598_2022_12249_MOESM1_ESM.pdf]

Development and validation of a nomogram to predict the risk of death within one year in patients with non-ischemic dilated cardiomyopathy：a retrospective cohort study

Yuan Huang[1,2,3] , Hai-Yan Wang[1,2,3], Wen Jian[1,2,3], Zhi-Jie Yang[1,2,3], Chun Gui[1,2,3]

[1]Department of Cardiology, The First Affiliated Hospital of Guangxi Medical University, Nanning 530021, Guangxi, People's Republic of China.

[2]Guangxi Key Laboratory Base of Precision Medicine in Cardio-Cerebrovascular Diseases Control and Prevention, Nanning 530021, Guangxi, People's Republic of China.

[3]Guangxi Clinical Research Center for Cardio-Cerebrovascular Diseases, Nanning 530021, Guangxi, People's Republic of China.

Corresponding author.

Correspondence to: Chun Gui, MD. Department of Cardiology, The First Affiliated Hospital of Guangxi Medical University, Nanning 530021, Guangxi, People's Republic of China. E-mail: guichun@stu.gxmu.edu.cn

Supplementary Table 1. Differences between demographic and clinical characteristics of the event and non-event groups.

demographic and clinical characteristics	died or underwent a heart transplant within one year		/ Z / /χ^2	*P*-Value
	No(n=510)	Yes(n=105)		
**General information**				
Gender , n(%)			0.051	0.821
Female	121 (23.7)	26 (24.8)		
Male	389 (76.3)	79 (75.2)		
Nationality , n(%)			1.504	0.220
Han nationality	255 (50)	60 (57.1)		
ethnic minorities	255 (50)	45 (42.9)		
Age(years)	55(46,64)	55(46,65)	0.037	0.971
Medical history, n(%)			17.554	<0.001
<1 year	264(51.8)	32(30.5)		
1-5 years	157(30.8)	41(39.0)		
≥5 years	89(17.5)	32(30.5)		
Heart failure diagnosed within the last 18 months, n(%)			14.051	< 0.001
No	188 (36.9)	60 (57.1)		
Yes	322 (63.1)	45 (42.9)		
Smoking history, n(%)			0.056	0.813
No	285(55.9)	60(57.1)		
Yes	225(44.1)	45(42.9)		
Current smoker, n(%)			0.377	0.539
No	391 (76.7)	84 (80)		
Yes	119 (23.3)	21 (20)		
Drinking history, n(%)			0.612	0.434
No	275(53.9)	61(58.1)		
Yes	235(46.1)	44(41.9)		
Pulmonary hypertension, n(%)			2.821	0.093
No	205(40.2)	33 (31.4)		
Yes	305 (59.8)	72 (68.6)		
Atrial fibrillation, n(%)			2.321	0.128
No	408 (80.0)	77 (73.3)		
Yes	102(20.0)	28 (26.7)		
History of stroke, n(%)			3.393	0.065
No	459(90.0)	88(83.8)		
Yes	51(10.0)	17(16.2)		
Diabetes, n(%)			1.087	0.297
No	472(92.5)	94(89.5)		
Yes	38(7.5)	11(10.5)		
Respiratory inflammation, n(%)			18.861	<0.001
No	334(65.5)	45(42.9)		
Yes	176(34.5)	60(57.1)		
COPD, n(%)			Fisher	1
No	496 (97.3)	102 (97.1)		
Yes	14 (2.7)	3 (2.9)		
Ventricular tachycardia/fibrillation, n(%)			1.538	0.215
No	473 (92.7)	93 (88.6)		

Yes	37 (7.3)	12 (11.4)		
Implantable cardiac devices, n(%)			Fisher	1
No	489 (95.9)	101 (96.2)		
Yes	21 (4.1)	4 (3.8)		
Grade of heart failure(NYHA), n(%)			19.787	<0.001
I	16(3.1)	1(1.0)		
II	108(21.2)	10(9.5)		
III	204(40.0)	34(32.4)		
IV	182(35.7)	60(57.1)		
**Physical examination**				
Body mass index(kg/㎡)	23.3(21.0,25.7)	22.2(19.7,23.8)	3.269	0.001
Heart rate(times/min)	88(76,100)	88(76,102)	0.451	0.652
Systolic pressure(mmHg)	116(104,128)	106(95,119)	4.506	<0.001
Diastolic pressure(mmHg)	76(68,86)	73(65,82)	1.921	0.055
Pulse pressure(mmHg)	38(30,49)	33(24,42)	3.635	<0.001
**Blood biochemical**				
NT-proBNP(pg/ml)	3242(1460,6692)	9207(4917,19300)	8.379	<0.001
White blood cell count($\times 10^9$/L)	7.3(6.1,9.0)	8.2(6.6,9.8)	2.933	0.003
Red blood cell count($\times 10^{12}$/L)	4.8(4.4,5.2)	4.5(4.1,4.9)	3.878	<0.001
Hemoglobin(g/L)	137(126,148)	132(119,142)	2.919	0.004
Platelet($\times 10^9$/L)	201(162,256)	188(148,242)	1.632	0.103
Neutrophil to Lymphocyte Ratio	2.4(1.7,3.7)	3.9(2.1,6.9)	5.129	<0.001
Neutrophils($\times 10^9$/L)	4.56(3.50,5.86)	5.50(4.22,7.44)	4.217	<0.001
Lymphocyte($\times 10^9$/L)	1.83(1.40,2.35)	1.55(1.03,1.99)	4.148	<0.001
Hematocrit	0.43(0.39,0.46)	0.41(0.37,0.44)	2.773	0.006
Prothrombin time(s)	12.5(11.1,13.4)	13.5(12.2,16.0)	5.788	<0.001
APTT(s)	32(30,34)	31(29,34)	0.809	0.419
Fibrinogen(g/L)	3.8(3.1,4.2)	3.7(2.8,4.3)	0.792	0.428
Thrombin time(s)	12.0(11.2,12.5)	12.0(10.9,12.9)	0.024	0.980
International normalized ratio	1.06(0.95,1.14)	1.15(1.03,1.34)	5.737	<0.001
Creatine kinase(U/L)	86(61,134)	98(55,173)	1.195	0.232
Creatine kinase-MB(U/L)	14(11,19)	16(12,24)	2.324	0.020
Lactic dehydrogenase(U/L)	247(203,301)	305(240,389)	6.054	<0.001
Lactic dehydrogenase-1(U/L)	69(54,90)	79(62,103)	2.929	0.003
α-HBD(U/L)	181(147,215)	211(175,273)	5.413	<0.001
Total cholesterol(mmol/L)	4.3(3.6,5.1)	4.0(3.4,4.5)	3.322	0.001
Triglycerides(mmol/L)	1.09(0.83,1.43)	0.98(0.78,1.29)	2.181	0.029
High-density lipoprotein(mmol/L)	1.00(0.80,1.18)	0.88(0.64,1.11)	3.219	0.001
Low-density lipoprotein(mmol/L)	2.67(2.11,3.21)	2.42(1.99,2.90)	2.196	0.028
Homocysteine(μmol/L)	15.2(12.0,18.1)	15.5(13.0,18.7)	0.776	0.438
Serum potassium (mmol/L)	4.0(3.7,4.4)	3.9(3.6,4.3)	1.810	0.070
Serum sodium(mmol/L)	139(137,141)	138(134,141)	2.757	0.006
Serum chlorine(mmol/L)	103(100,106)	101(95,105)	4.232	<0.001
Albumin(g/L)	38.8(35.6,41.8)	37.6(34.2,40.4)	2.870	0.004
Globulin(g/L)	26(23,30)	27(22,30)	0.189	0.850
Albumin to Globulin Ratio	1.5(1.3,1.7)	1.4(1.2,1.7)	1.395	0.163
Aspartate aminotransferase(U/L)	31(23,42)	40(26,69)	4.053	<0.001
Alanine aminotransferase(U/L)	30(19,52)	32(20,69)	1.390	0.165
Urea(mmol/L)	6.8(5.4,8.7)	8.3(6.1,11.6)	4.205	<0.001

Creatinine(μmol/L)	92(77,110)	102(82,128)	2.856	0.004
Glomerular filtration rate(mL/min)	66.5(51.9,88.1)	56.1(40.1,76.6)	3.964	<0.001
Cystatin C(mg/L)	1.04(0.84,1.28)	1.17(0.94,1.57)	3.557	<0.001
Uric acid(μmol/L)	477(377,600)	521(423,667)	2.467	0.014
**Echocardiographic**				
LAD(mm)	45(41,51)	46(42,51)	1.320	0.187
LVDd(mm)	68(63,74)	73(67,79)	4.265	<0.001
End-diastolic volume(ml)	238(196,291)	270(220,328)	3.845	<0.001
LVFS, n(%)	16(13,20)	15(12,18)	2.225	0.026
LVEF, n(%)	33(27,40)	30(26,36)	2.309	0.021
Stroke volume(ml/B)	77(59,96)	84(65,104)	1.873	0.061
Cardiac output(L/min)	6.8(5.2,8.6)	7.4(5.4,9.1)	1.428	0.153
**Treatment and drug**				
in-hospital worsening heart failure, n(%)			72.798	<0.001
No	500(98.0)	81(77.1)		
Yes	10(2.0)	24(22.9)		
Dopamine Injection, n(%)			48.333	<0.001
No	415(81.4)	52(49.5)		
Yes	95(18.6)	53(50.5)		
Nitroglycerin Injection, n(%)			11.290	0.001
No	310(60.8)	82(78.1)		
Yes	200(39.2)	23(21.9)		
Diuretics, n(%)			0.253	0.615
No	8(1.6)	3(2.9)		
Yes	502(98.4)	102(97.1)		
MRA			4.117	0.042
No	186 (36.5)	50 (47.6)		
Yes	324 (63.5)	55 (52.4)		
Beta blockers, n(%)			7.433	0.006
No	57(11.2)	22(21.0)		
Yes	453(88.8)	83(79.0)		
ACEIs or ARBs, n(%)			34.327	<0.001
No	65(12.7)	38(36.2)		
Yes	445(87.3)	67(63.8)		

Notes: The calculation formula of glomerular filtration rate is as follows: Male: glomerular filtration rate (ml/min) = ((140-age)×weight (kg))×1.23÷serum creatinine (μmol/L); Female: glomerular filtration rate (ml/min) =(140-age)×weight (kg)×1.04÷serum creatinine (μmol/L).

Abbreviations: APTT, Activated partial thromboplastin time; α-HBD, Alpha-hydroxybutyrate dehydrogenase; ACEIs, Angiotension converting enzyme inhibitors; ARBs, Angiotensin Receptor Blockers; COPD, Chronic obstructive pulmonary disease; LAD, Left atrium anteroposterior dimension; LVDd, Left ventricular end diastolic dimension; LVFS, Left ventricular fractional shortening; LVEF, Left ventricular ejection fraction; MRA, Mineral corticoid receptor antagonist; NT-proBNP, N terminal pro B type natriuretic peptide; NYHA, New York Heart Association.

Supplementary Table 2. The best cut-off point values for the ten continuous variables identified after LASSO regression.

**Variables**	**AUC**	**Youden Index(maximum)**	**Cut-off point(corresponding)**	**Cut-off point(final)**
Body mass index(kg/m²)	0.601	0.174	23.9	24
Systolic pressure(mmHg)	0.639	0.230	110	110(mmHg)
Pulse pressure	0.613	0.177	35(mmHg)	35(mmHg)
Red blood cell count	0.620	0.215	4.6($\times 10^{12}$/L)	4.5($\times 10^{12}$/L)
Total cholesterol	0.603	0.192	4.2(mmol/L)	4.2(mmol/L)
Serum chlorine	0.631	0.229	96(mmol/L)	96(mmol/L)
Neutrophil to Lymphocyte Ratio	0.659	0.280	3.9	4.0
International normalized ratio	0.677	0.282	1.1	1.0
Aspartate aminotransferase	0.625	0.224	39(U/L)	40(U/L)
NT-proBNP	0.763	0.409	5390(pg/ml)	5400(pg/ml)
LVDd	0.632	0.220	71(mm)	70(mm)

Notes: Receiver-operating characteristic (ROC) curve analysis was performed to determine the optimal cutoff point value of the ten indexes.

Abbreviations: NT-proBNP, N terminal pro B type natriuretic peptide; LVDd, Left ventricular end diastolic dimension; AUC,area under the curve.
